# Heparanase augments insulin receptor signaling in breast carcinoma

**DOI:** 10.18632/oncotarget.14292

**Published:** 2016-12-27

**Authors:** Rachel Goldberg, Amir Sonnenblick, Esther Hermano, Tamar Hamburger, Amichay Meirovitz, Tamar Peretz, Michael Elkin

**Affiliations:** ^1^ Sharett Institute, Hadassah-Hebrew University Medical Center, Jerusalem 91120, Israel

**Keywords:** heparanase, insulin receptor, breast carcinoma, diabetes

## Abstract

Recently, growing interest in the potential link between metabolic disorders (i.e., diabetes, obesity, metabolic syndrome) and breast cancer has mounted, including studies which indicate that diabetic/hyperinsulinemic women have a significantly higher risk of bearing breast tumors that are more aggressive and associated with higher death rates. Insulin signaling is regarded as a major contributor to this phenomenon; much less is known about the role of heparan sulfate-degrading enzyme heparanase in the link between metabolic disorders and cancer.

In the present study we analyzed clinical samples of breast carcinoma derived from diabetic/non-diabetic patients, and investigated effects of heparanase on insulin signaling in breast carcinoma cell lines, as well as insulin-driven growth of breast tumor cells.

We demonstrate that heparanase activity leads to enhanced insulin signaling and activation of downstream tumor-promoting pathways in breast carcinoma cells. In agreement, heparanase enhances insulin-induced proliferation of breast tumor cells *in vitro*. Moreover, analyzing clinical data from diabetic breast carcinoma patients, we found that concurrent presence of both diabetic state and heparanase in tumor tissue (as opposed to either condition alone) was associated with more aggressive phenotype of breast tumors in the patient cohort analyzed in our study (two-sided Fisher's exact test; p=0.04). Our findings highlight the emerging role of heparanase in powering effect of hyperinsulinemic state on breast tumorigenesis and imply that heparanase targeting, which is now under intensive development/clinical testing, could be particularly efficient in a growing fraction of breast carcinoma patients suffering from metabolic disorders.

## INTRODUCTION

Epidemiological evidence clearly indicates increased association between breast carcinoma and metabolic disorders (i.e., obesity; type 2 diabetes, metabolic syndrome) [1–5]. In fact, numerous studies have confirmed a higher risk of bearing breast tumors as well as unfavorable breast cancer prognosis in obese, diabetic and hyperinsulinemic patients (reviewed in [6]). While the relative contribution of metabolic alterations versus the direct effects of increased adipose tissue (e.g., adipokine/cytokine secretion, augmented production of estrogens) on breast tumor promotion have been greatly debated [3], extensive research in the field clearly points to the crucial role of hyperinsulinemia and insulin signaling [2, 3, 6–8]. Indeed, the aforementioned metabolic conditions are manifested by an increase in circulating insulin [3], since under state of insulin resistance (a central feature in the aetiology of type 2 diabetes, most frequently caused by obesity) the pancreatic β cells induce a physiological compensatory response that results in hyperinsulinaemia. Tumor promoting activity of insulin can be mediated by insulin receptor (INSR) as well as cross-activation of the insulin-like growth factor 1 receptor (IGF1R) family, resulting in activation of downstream signaling cascades [3, 7]. In particular, the phosphatidylinositol-3 kinase (PI3K)/AKT pathway is a critical component of the insulin responses, promoting breast tumor cell growth, survival and aggressive behaviour. Its importance has recently been highlighted by the fact that 50% of breast carcinomas have an activated PI3K/AKT pathway due to mutations in one of its components [9]. Of note, hyperinsulinemia increases mammary tumor growth *in vivo*, and blockade of the INSR/IGF1R reduces tumor growth in hyperinsulinemic mice [7]. Moreover, most recent clinical observations revealed that breast carcinoma patients with low IR expression levels had significantly longer progression-free survival and overall survival [10], while presence of phosphorylated INSR/IGF1R is related to poor survival in all breast cancer subtypes [11]. Extracellular matrix (ECM) components, such as heparan sulfate (HS) proteoglycans, and their enzymatic remodeling may also affect insulin-INSR signaling axis [12–15], however direct contribution of these factors to accelerated breast tumor progression/aggressiveness in patients with metabolic disorders remained underappreciated.

Here we present the evidence that activity of heparanase enzyme (the only known mammalian endoglycosidase that cleaves HS) leads to enhanced insulin signaling and activation of downstream tumor-promoting pathways in breast carcinoma cells.

HS proteoglycans are ubiquitously found in the ECM and plasma membrane of cells: HS polysaccharide chains bind to and assemble ECM proteins, playing important roles in the integrity of the ECM [16, 17]. In addition, HS chains regulate the activity of a wide variety of bioactive molecules (i.e., cytokines, growth factors) at the cell surface and in the ECM [16, 18]. Enzymatic degradation of HS by heparanase profoundly affects numerous pathophysiological processes, including cell growth, differentiation and motility [14, 19–21]. Since uncontrolled cleavage of HS could result in significant tissue damage, under normal conditions heparanase is kept tightly regulated: with a few exceptions (placenta, activated immune cells), in non-cancerous cells/tissues heparanase promoter is inhibited and the gene is not expressed. Notably, upregulation of heparanase was reported in various cancer types [14, 19–21]. During tumor progression, the enzyme was reported to contribute, among other aspects, to the breakdown of extracellular barriers, bioavailability of HS-binding growth factors and generation bioactive HS fragments potentiating growth factor-receptor binding/signaling [18, 20–22].

Preferential expression and involvement of heparanase in breast cancer are particularly well documented, both in experimental [19, 23–26] and clinical settings [27, 28]. Importantly, increased heparanase levels are associated with reduced patients’ survival post operation and increased tumor aggressiveness [14, 21]. Moreover, heparanase expression in several tumor types (including breast) was linked to therapy resistance [28, 29].

Altogether, these data, along with the recently reported ability of the enzyme to enhance INSR signaling in myeloma [15], prompted us to hypothesize that heparanase may play an important role in diabetes-associated breast cancer, facilitating tumor aggressiveness under hyperinsulinemic state. In the present study we report that in breast carcinoma patients, diabetic state conferred more aggressive phenotype (manifested by increased lymph node involvement) to heparanase-positive, as compared to heparanase-negative tumors. In agreement with clinical observations, *in vitro* heparanase enzyme augmented INSR signaling / downstream AKT activation in breast carcinoma cell lines, and enhanced insulin-induced growth of breast tumor cells. Taken together, our findings highlight the emerging role of heparanase in modulating pro-tumorous effect of hyperinsulinemic state on breast tumorigenesis and imply that heparanase-targeting therapeutic approaches could be particularly beneficial in breast carcinoma patients suffering from metabolic disorders.

## RESULTS

### Correlation between heparanase expression and lymph node involvement in diabetic breast carcinoma patients

**Figure 1:**
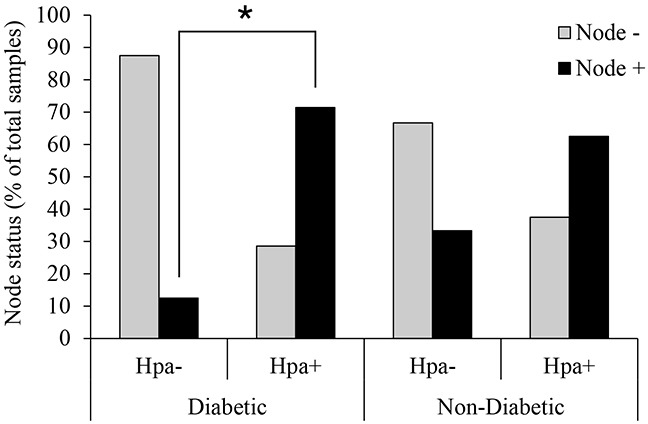
Heparanase expression and lymph node involvement in diabetic breast carcinoma patients Human breast carcinoma tissue samples (biopsies) were processed for immunohistochemistry with anti-heparanase antibody (733) directed against a synthetic peptide (^158^KKFKNSTYRSSSVD^171^) corresponding to the N-terminus of the 50-kDa subunit of the heparanase enzyme, as described in [28, 57, 58]. Diabetic state, BMI and lymph node status were determined from patient history. Node+: patients with lymph node positive tumors; Node-: patients with lymph node negative tumors. Two-sided Fisher's exact test confirmed that in diabetic patients heparanase-positive tumors are more likely to spread into lymph nodes (*p=0.04). Presence of heparanase in non-diabetic breast tumor samples did not confer statistical significant difference in lymph node involvement.

Women with diabetes have higher breast cancer incidence and mortality, exhibiting a higher risk of lymph node metastases [4, 5]. Therefore, to explore the hypothesized role of heparanase in diabetes-breast cancer link, we first examined correlation between expression of heparanase in tumor tissue and lymph node involvement, using clinical data from 67 breast carcinoma patients (15 with diabetes mellitus and 52 non diabetic/non-obese controls). Since obesity is the main cause of insulin resistance and in many individuals is the first step in the development of type 2 diabetes and metabolic syndrome [3, 30], obese patients were excluded from the non-diabetic control group. Among diabetic patients bearing heparanase-positive tumors, more than 70% (5 out of 7) displayed lymph node involvement. In contrast, in only 12.5% diabetic patients with heparanase-negative tumors (one out of eight) node involvement was noted. Statistical analysis confirmed that under diabetic state heparanase-positive tumors are more likely to spread into lymph nodes (two-sided Fisher's exact test; p=0.04; Figure 1). Of note, presence of either diabetic state or heparanase alone did not confer statistical significant difference in lymph node involvement (Figure 1).

### Heparanase enhances INSR signaling and insulin-induced proliferation in breast carcinoma cells

The above clinical observations (although limited by a small sample size), along with importance of insulin-INSR signaling in diabetes/obesity-related cancer [2, 3, 6–8] and the recent report on heparanase-augmented INSR signaling in myeloma [15], lead us to hypothesize that heparanase facilitates breast carcinoma progression under diabetic conditions via augmentation of INSR signaling.

**Figure 2:**
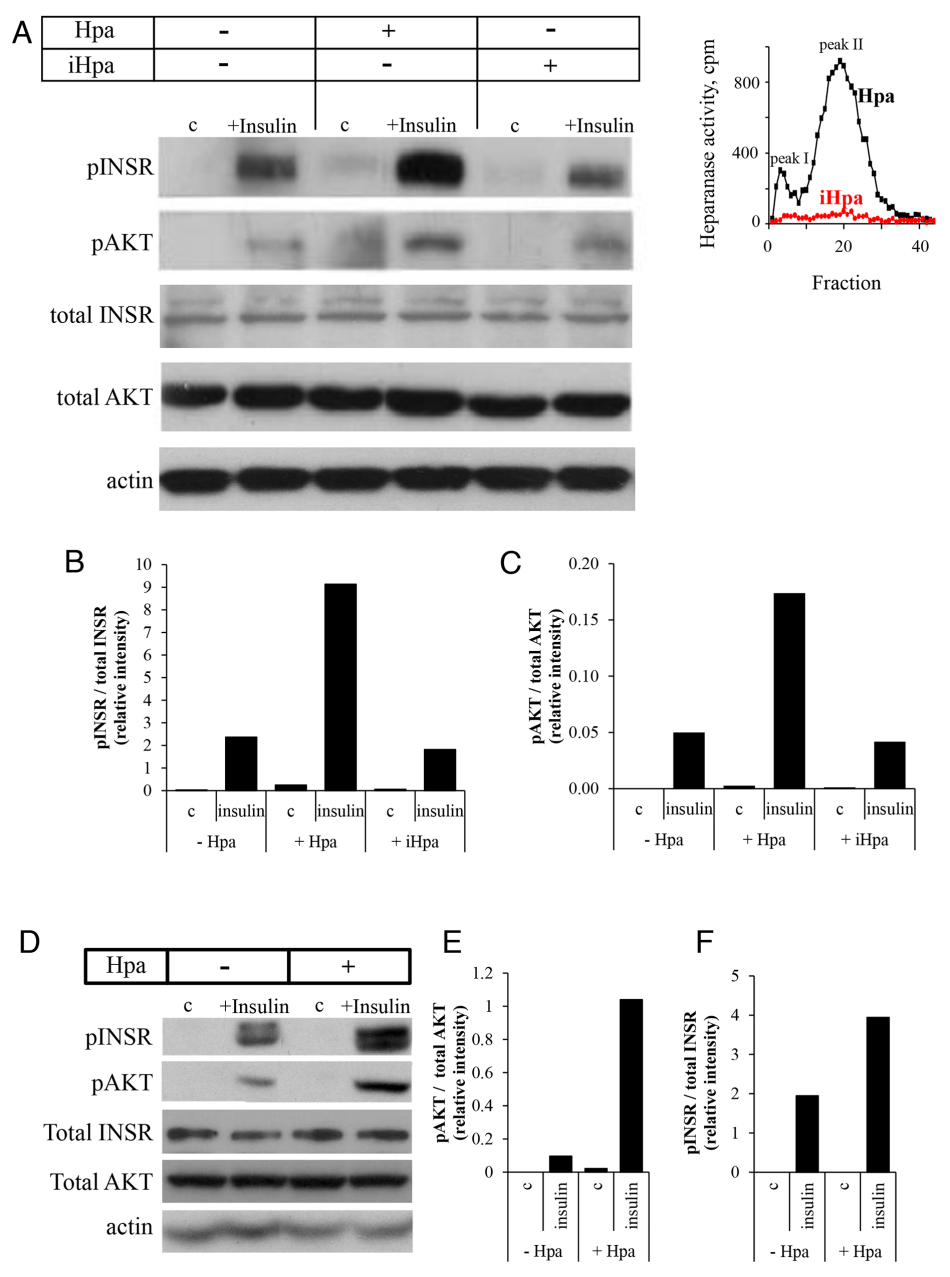
Recombinant heparanase enzyme enhances insulin receptor signaling pathway in breast cancer cells **A–C**. Human breast carcinoma MCF-7 cells were serum-starved overnight and then either remained untreated (c) or stimulated with insulin (100 nM) for 30 min in the absence or presence of 0.8 μg/ml active recombinant heparanase (Hpa) or heat-inactivated heparanase (iHpa). **A**. Cell lysates containing equivalent amounts of total protein were then immunoblotted using antibody specific for phospho-insulin receptor (pINSR), phospho-AKT (pAKT), total INSR, total AKT or total actin. **B, C**. The band intensity was quantified using ImageJ software; intensity ratio for pINSR/total INSR (B) and pAKT/total AKT (C) are shown. The data are representative of three independent experiments. **Inset**: Enzymatic activity in samples of Hpa (black line) and iHpa (red line) was examined as described in Methods. **D–F**. Mouse E0771 breast cancer cells (characterized by low endogenous levels of heparanase) were serum-starved overnight and then treated as described in **A-C**. Intensity ratio for pINSR/total INSR (E) and pAKT/total AKT (F) are shown. The data are representative of three independent experiments.

To test this hypothesis, we first incubated ER-positive MCF-7 human breast carcinoma cells with insulin in the absence or presence of recombinant active heparanase enzyme. As expected, INSR signaling was induced following treatment with insulin, as evident by increased phospho-INSR (pINSR) and phospho-AKT (pAKT) levels (Figure 2A-2C). However, presence of recombinant heparanase significantly increased pINSR and pAKT levels in response to insulin treatment (Figure 2A-2C). Protein levels of total AKT and total INSR were not affected by presence of heparanase. Notably, this augmented response to insulin in the presence of heparanase was dependent on heparanase enzymatic activity, since treatment with heat-inactivated heparanase (iHpa, Figure 2 inset) did not affect pINSR/pAKT levels (Figure 2). Similar results were obtained in ER-positive E0771 murine breast carcinoma cells (Figure 2D-2F). Of note, presence of recombinant heparanase also augmented INSR signaling in ER-negative breast carcinoma cell line MDA-MB-231 (2-fold increase in pINSR levels, not shown), consistent with the reports that progression of both hormone dependent and independent breast tumors is affected by INSR signaling [31, 32].

**Figure 3:**
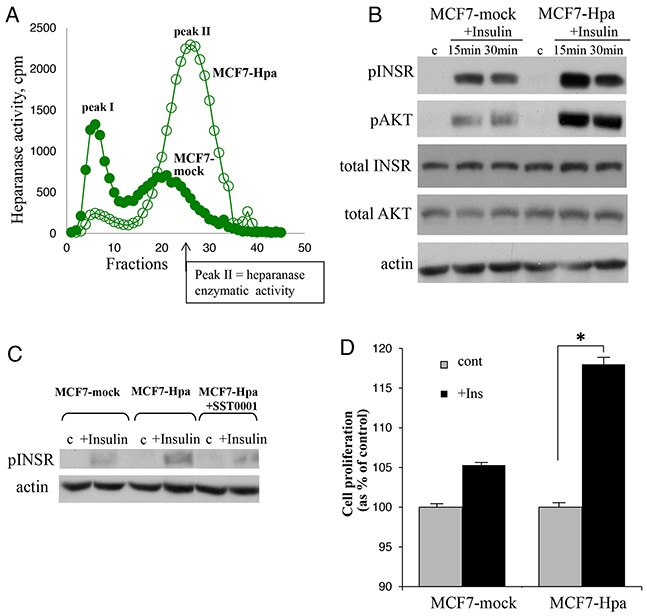
Heparanase overexpression enhances insulin receptor signaling pathway and augments proliferative response to insulin in MCF-7 cells **A**. Heparanase overexpression in MCF7-Hpa (as compared to control MCF7-mock) cells was confirmed by activity assay, as described in Method and refs.(19, 54). **B**. MCF7-mock and MCF7-Hpa cells were serum-starved overnight and then either remained untreated (c) or stimulated with insulin (100 nM) for 15 and 30 min. Cell lysates containing equivalent amounts of total protein were then immunoblotted using antibody specific for pINSR, pAKT, total INSR, total AKT or total actin. The data shown are representative of three independent experiments. **C**. Presence of heparanase specific inhibitor SST0001 (10 μg/ml) reduced pINSR levels in MCF7-Hpa cells treated with insulin. **D**. Bar graph demonstrates the increase in proliferation of MCF7-mock and MCF7-Hpa cells cultured for 72 h in the absence (gray bars) or presence of insulin (100 nM, black bars), analyzed by MTS Cell Proliferation Assay. Note that presence of insulin does not confer statistically significant increase in proliferation of MCF7-mock cells (lacking heparanase activity). In contrast, in heparanase overexpressing MCF7-Hpa cells proliferation rate was significantly higher in the presence of insulin. *p<0.04 Two-sided Student's *t* test. Error bars represent ± SD. The data shown are representative of three independent experiments.

We then utilized MCF-7 cells stably transfected with vector encoding for human heparanase (*MCF7-Hpa*) or mock-transfected with empty vector (*MCF7-mock*) (Figure 3A). As shown in Figure 3B, treatment with insulin resulted in significantly increased pINSR and pAKT levels in MCF7-Hpa cells as compared to MCF7-mock cells. This effect was abolished in the presence of specific inhibitor of heparanase SST0001 (Figure 3C). Protein levels of total AKT and total INSR were not affected by presence of heparanase (Figure 3B). Next, to test the biological consequence of heparanase-augmented INSR signaling, we compared proliferation rate of MCF7-Hpa and MCF7- mock cells in response to insulin treatment. As shown in Figure 3D, proliferation in response to insulin was markedly enhanced in MCF7-Hpa, as compared to MCF7-mock cells. Altogether these results demonstrate that INSR signaling in breast carcinoma cells is enhanced in the presence of heparanase and results in accelerated cell proliferation.

## DISCUSSION

It is well recognized that hyperinsulinemia and INSR signaling are critical determinants responsible for accelerated progression and aggressive phenotype of breast cancer in patients with metabolic disorders (i.e., diabetes, obesity) [2, 3, 6–8]. Heparanase enzyme has also been implicated in breast tumor progression [19, 23–28]. In the present study we show that the interplay between heparanase and insulin signaling may foster breast tumorigenesis, mechanistically linking the enzyme into the breast cancer-promoting action of metabolic disorders. Underscoring this previously unrecognized role of the enzyme in this phenomenon, we found statistically significant association between lymph node involvement and simultaneous presence of both diabetic state and heparanase expression (two-sided Fisher's exact test; p=0.04; Figure 1). Of note, diabetic state alone or heparanase overexpression alone were not associated with statistically significant increase in lymph node involvement in the patient cohort analyzed in our study (Figure 1). In agreement with our clinical observations is the ability of heparanase to augment insulin-induced proliferation in breast carcinoma cells *in vitro* (Figure 3D), particularly relevant in light of the previous reports showing association between high proliferative rate of breast carcinoma cells and lymph node metastasis [33, 34].

While our findings reveal augmented insulin receptor signaling in the presence of heparanase in breast carcinoma, a limitation of our study is that precise molecular mechanism underlying this phenomenon has not been determined. One intriguing possibility is that soluble heparan sulfate, liberated by heparanase, may facilitate formation and stabilization of insulin-INSR signaling complexes. Indeed, it was previously demonstrated that heparin or heparan sulfate proteoglycans (including HS fragments generated by heparanase enzyme [22]) function as growth factor tyrosine kinase receptor accessory molecules, ligands or co-ligands [35–41], potentiate ligand-receptor binding, dimerization, and signaling. Of note, the cell surface HS proteoglycan syndecan-1 coupled ternary receptor complex (prevalent on tumor cells and activated endothelial cells) has been described, whereby syndecan-1 clusters IGF-1R and integrins, leading to integrin activation [42, 43]. In a similar manner, heparanase-released bioactive HS fragments could stimulate the clustering with INSR leading to its dimerization and autophosphorylation. Additionally, a recent study demonstrated that cell surface heparan sulfate proteoglycans (i.e., glypicans), are capable of interacting with and enhancing INSR signaling [12, 13]. The effect on INSR signaling appears to involve release of glypican from the cell surface of adipocytes and possibly other cell types by an enzymatically regulated process and its direct interaction with INSR [13], likely through heparan sulfate chains. This mode of action could be particularly relevant in light of a previously demonstrated role of heparanase enzymatic activity in shedding of cell surface heparan sulfate proteoglycans [14], and release of cell surface-derived bioactive heparan sulfate that potentiates growth factor-receptor signaling [18, 22].

Our *in vitro* studies demonstrated ability of heparanase to enhance INSR and perhaps IGF1R signaling in both ER -positive (MCF-7, E0771) and -negative (MDA-MB-231) cell lines. Yet, ER status could still be an important factor in inducing heparanase expression in breast tumors, therefore contributing to the occurrence of INSR-heparanase interplay under diabetic state. Estrogen signaling has been previously shown to induce heparanase in breast carcinoma [28, 44]. A frequent accompanying feature of type 2 diabetes is obesity, and obese state is characterizes by elevated estrogen levels in women [due to increased aromatase expression [1, 45, 46]. Thus, estrogen is expected to upregulate heparanase in breast tumor cells *per se* [44]. At the same time, several diabetic milieu constituents, known to increase heparanase expression/secretion in endothelial cells and immunocytes [47–51], may augment the expression of the enzyme in stromal elements of the tumor, suggesting that a self-sustaining circuit may exist in metabolic disorder-related ER positive breast cancer, where estrogen, and components of the diabetic milieu induce heparanase, while heparanase acts in tandem with elevated insulin to promote cell growth via enhanced INSR signaling. Noteworthy, reports by Parish and co-workers also suggested a role for heparanase in the pathogenesis of diabetes *per se* [52, 53].

Although further studies (including analysis of larger patient groups) are warranted to fully dissect the complex molecular events underlying action/regulation of heparanase in modulating effects of metabolic disorders on breast cancer progression, our findings point to the importance of the interplay between enzymatic activity of heparanase and insulin signaling in facilitating breast tumorigenesis. Moreover, our observations imply that heparanase-targeting therapeutic approaches, which are now under intensive development/clinical testing [14], alone or in combination with INSR/IGF1R pathway inhibition, may disrupt this interplay and therefore be particularly beneficial in a significant fraction of breast cancer patients.

## MATERIALS AND METHODS

### Cell culture and transfection

MCF-7 and MDA-MB-231 human breast carcinoma cells and E0771 mouse breast carcinoma cells were grown in RPMI 1640 medium supplement with 1 mM glutamine, 50 μg/ml streptomycin, 50 U/ml penicillin and 10% fetal calf serum (FCS) (Biological Industries) at 37°C and 8% CO_2_. For stable transfection, MCF-7 cells were transfected with human heparanase cDNA (H-*hpa* transfectants) or with a control empty pcDNA3 vector (Invitrogen) (mock transfectants), as previously described [28]. Transfected cells were selected with 800 μg/ml G418 and stable populations of heparanase expressing cells were obtained. To rule out the possibility of insertional mutagenesis, all the experiments involving heparanase- and mock-transfected cells have been conducted using a pooled population of clones, which contained over 100 clones mixed together. Expression of heparanase was evaluated by RT-PCR and verified by measurements of enzymatic activity, as described below and in refs. [19, 25, 54, 55].

Prior to insulin treatment 60-80% confluent cells, maintained overnight in serum-free RPMI, remained untreated or were incubated with 100nM insulin (Biological Industries) for 15 or 30 minutes. Cells were then lysed and processed for western blot analysis.

### Antibodies

Immunoblot analysis was carried out with the following antibodies: anti-phospho-insulin receptor Tyr1150/1151, anti-phospho-AKT Ser 473, anti-total insulin receptor, anti- total AKT (Cell Signaling) and anti-actin (Abcam).

### Immunoblotting

MCF-7 whole cell lysates were homogenized in lysis buffer containing 0.6% SDS, 10 mM Tris-HCl, pH 7.5, supplemented with a mixture of protease inhibitors (Roche) and phosphatase inhibitors (Thermo Scientific). Equal protein aliquots were subjected to SDS-PAGE (8% acrylamide) under reducing conditions and proteins were transferred to a polyvinylidene difluoride membrane (Millipore). Membranes were blocked with 3% BSA for 1 hour at room temperature and probed with the appropriate antibody, followed by horseradish peroxidase-conjugated secondary antibody (KPL) and a chemiluminescent substrate (Biological Industries).

### Heparanase activity assay

Measurements of heparanase enzymatic activity was carried out as described previously [19, 54, 55]. Briefly, tested samples were incubated (16-36 h, 37°C, pH 6.2) on dishes coated with sulfate-labeled ECM, prepared as described [19, 54, 55]. Sulfate-labeled material released into the incubation medium was analyzed by gel filtration on a Sepharose 6B column. Nearly intact heparan sulfate proteoglycans are eluted just after the void volume (peak I, K_av_ < 0.2, fractions 1-10). This material (peak I) has been previously shown to be generated by a proteolytic activity residing in the ECM itself; Heparan silfate (HS) degradation fragments are eluted later with 0.5<K_av_<0.8 (peak II, fractions 15-35) [19, 54]. These fragments were shown to be degradation products of HS as they were 5-6 fold smaller than intact HS side chains, resistant to further digestion with papain and chondroitinase ABC, and susceptible to deamination by nitrous acid [19, 54]. Each experiment was performed at least three times and the variation in elution positions (*K*_av_ values) did not exceed ±15%.

### MTS assay

MCF-7 cells were seeded in 96-well culture plates in serum free RPMI. MTS assay (Promega) was performed according to manufacturer instructions and proliferation was measured 72 hours after insulin (100 nM) was added. Each experiment was performed at least 3 times. Each data point shows the mean of pentaplicate cultures.

### Clinical data and immunostaining

Data from 67 breast carcinoma female patients (15 diabetic and 52 non-diabetic with BMI<30) were available from the Sharett Oncology Institute, Hadassah Medical Center, Jerusalem. The use of these data and formalin-fixed, paraffin-embedded breast carcinoma tissues in research was approved by the Human Subjects Research Ethics Committee of the Hadassah Medical Center. The collective median age of the patient cohort was 50 years (range 20–82). Diabetes history was obtained from the clinical charts forms and determined using the medical history coded WHO-DDE - World Health Organization medical dictionaries. Patients for whom diabetic history/diabetic treatment drugs were found upon review of the clinical charts were included in the diabetic group, otherwise the patients were included in the control group. Determination of heparanase expression status: five-micron sections of tumor tissue were deparaffinized and rehydrated. Tissue was then incubated in 3% H_2_O_2_, denatured by boiling (3 min) in a microwave oven in citrate buffer (0.01 M, pH 6.0), and blocked with 10% goat serum in PBS. Sections were incubated with polyclonal rabbit anti-heparanase antibody (733) directed against a synthetic peptide (^158^KKFKNSTYRSSSVD^171^) corresponding to the N-terminus of the 50-kDa subunit of the HPSE enzyme [56]. The antibody was diluted 1:100 in 10% goat serum in PBS. Control slides were incubated with 10% goat serum alone. Color was developed as described in [28] and slides were visualized with a Zeiss axioscope microscope and manually read by an expert pathologist. To define tumor as heparanase-positive, a cutoff point of 25% immunostained tumor cells was chosen on the basis of an initial overview of the cases, in order to improve signal-to-noise ratios. Cutoff was chosen before any attempt at correlating heparanase expression with lymph node involvement.

### Statistical analysis

The results are presented as the mean ±SD. P values ≤0.05 were considered statistically significant. Statistical analysis was performed by unpaired Student's t-test unless otherwise stated. Fisher's exact test was performed to study the relationship between heparanase immunohistochemical results and clinical parameters. All statistical tests were two-sided.
